# Excision of the Right-Superior Maxilla, and of a Considerable Portion of the Left, for a Tumor of the Antrum

**Published:** 1864-08

**Authors:** E. R. Mordecai

**Affiliations:** Mobile, Alabama.


ART. IV.—*Excision of the Right-Superior Maxilla, and of a considerable portion of the Left, for a Tumor of the Antrum.* By E. R. MORDECAI, M. D., Mobile, Alabama.

Before the war, it was regarded as legitimate to report surgical cases occurring in general practice. Whether your journal, sprung from the novel exigences of the times in which we live, has adopted such rules as will admit the following communication into its pages, it remains, Mr. Editor, for you to determine.

In the latter part of February, 1862, Mary, aged eighteen, apparently of good constitution, was sent to me for examination by her master, Dr. J. H. Hastie, of Baldwin county, of this State (Alabama.)

The right side of the face was greatly deformed, by an enormous enlargement of the upper jaw, forming a tumor over which the skin and muscular tissue of the cheek were found to be movable. The teeth were sound, but many of them loose in their sockets. The alveolar process was enlarged and softened from the third molar tooth of the right-superior maxilla, to the first molar of the corresponding bone on the opposite side.

The walls of the antrum on the right side were softened, expanded, and projected forward and upward, so as partially to close the eye. The entire nasal process on the right side, and the inferior portion of that of the left, were altered by the disease.

An exploring needle was passed through the softened walls of the antrum, and a small quantity of discolored liquid withdrawn. The instrument did not move freely within the antrum, which seemed to be blocked up with a pulpy mass. A few days after this examination, a fungous growth shot forth from the point of exploration, grew rapidly, and bled freely when touched.

From the history of the case, it was ascertained that the disease was of four years' standing; that the morbid movement, slow at first, had advanced rapidly in the last six months of its duration. There could be no doubt about the nature of the affection. It was an encephaloid tumor of the upper jaw, which, originating in the antrum of the right maxilla, had involved the whole of that bone; and, crossing the median line, had affected the alveolar, palate, and nasal processes of the opposite side.

The patient being very anxious for an operation, and the sub-maxillary and other glands appearing to be in a normal condition, it was determined to yield to her solicitations. She was sent to the Providence Infirmary, of this city (Mobile) where, on the 10th of March, 1862, aided by Drs. Gaines and Cox, and by the assistants of the Infirmary, the following operation was made:

It was thought that, by free dissection, the entire mass of disease could be removed through Velpeau's simple curvilinear incision. This was made through the cheek on the right side, and extended from the middle of the zygoma, to the commissure of the lip. After dissecting up this flap to the orbit, and freeing the globe of the right eye from its inferior and lateral attachments, the dissection was carried through the frœnum of the lip and cartilages of the nose, to the left side of the face, so as to uncover the diseased portions of the left maxilla.

With Liston's cutting forceps, the osseous connections were then severed, and the whole of the right-superior maxilla, with the malar bone, was excised. Of the left maxilla, the alveolar process as far back as the first molar tooth, two-thirds of the palate process, and the inferior portion of the nasal process, together with the greater part of the vomer, were removed.

The cut surfaces were brought together and maintained in apposition by silver-wire and adhesive strips. The cheek was *not* stuffed with lint, as is the fashion in Europe; but had a simple cold-water dressing applied externally.

My patient entirely recovered. Ten days after the operation she was walking about, as if nothing unusual had occurred—the long line of incision in her cheek having nearly healed by first intention. In the course of a week or two she returned home, where she now is, actively employed about her master's household, as she has been since her recovery from the operation in 1862.

